# Energizing Healing with Electromagnetic Field Therapy in Musculoskeletal Disorders

**DOI:** 10.26502/josm.511500147

**Published:** 2024-05-17

**Authors:** Resmi Rajalekshmi, Devendra K Agrawal

**Affiliations:** Department of Translational Research, College of the Osteopathic Medicine of the Pacific, Western University of Health Sciences, Pomona, California USA

**Keywords:** Connective tissue disorder, Electromagnetic field, Pulsed electromagnetic field, Musculoskeletal system, Healthcare, Intracellular signaling, Tendon healing, Therapy of musculoskeletal disorders

## Abstract

There is mounting evidence to suggest that exogenous electromagnetic fields (EMF) may play a significant role in various biological processes that are crucial to therapeutic interventions. EMFs have been identified as a non-invasive, safe, and effective therapy that appears to have no apparent side effects. Numerous studies have demonstrated that pulsed EMFs (PEMFs) have the potential to become a stand-alone or adjunctive treatment modality for managing musculoskeletal disorders. However, several questions remain unresolved. Before their widespread clinical application, further research from well-designed, high-quality studies is required to standardize treatment parameters and determine the optimal protocol for healthcare decision-making. This article provides a comprehensive overview of the impact of musculoskeletal diseases on overall well-being, the limitations of conventional treatments, and the need to explore alternative therapeutic modalities such as electromagnetic field (EMF) therapy. EMF therapy uses low-frequency electromagnetic waves to stimulate tissue repair, reduce inflammation, and modulate pain signals, making it a safe and convenient alternative to conventional treatments. The article also discusses the historical perspective of EMF therapy in medicine. The article highlights the potential of EMF therapy as a personalized and comprehensive care option for musculoskeletal diseases, either alone or in conjunction with other therapies. It emphasizes the imperative for further research in this field and presents a compelling case for the use of EMF therapy in managing musculoskeletal diseases. Overall, the available findings on the underlying cellular and molecular biology support the use of EMF therapy as a viable option for the management of musculoskeletal disorders and stresses the need for continued research in this area.

## 1. Introduction

### 1.1 Brief overview of musculoskeletal diseases

The human musculoskeletal system, comprised of bones, muscles, joints, ligaments, tendons, and connective tissues, is integral to providing structural support, enabling movement, and safeguarding vital organs [1]. Its proper functioning is fundamental for overall well-being and contributes to metabolic processes [2].

The Global Burden of Diseases, Injuries, and Risk Factors Study (GBD) identifies five specific musculoskeletal conditions: rheumatoid arthritis, osteoarthritis, low back pain, neck pain, and gout [3]. Additionally, a broader category termed “other musculoskeletal disorders” encompasses various acute and chronic conditions affecting the locomotor and connective tissue systems. This heterogeneous group includes spondyloarthropathies, inflammatory arthritis (excluding rheumatoid arthritis), vasculitis, autoimmune conditions like systemic lupus erythematosus, chronic musculoskeletal pain syndromes such as fibromyalgia, osteopathies, chondropathies, disorders of bone density and structure, as well as disorders of synovium, tendons, and connective tissue. The category also encompasses other undefined disorders of the musculoskeletal system and connective tissue not explicitly modeled in the GBD.

These musculoskeletal diseases extend beyond physical discomfort, leading to functional limitations, reduced mobility, and a diminished quality of life [4]. Diagnosing and managing these conditions involve collaboration across medical disciplines, including rheumatology, orthopedics, and physical therapy.

Preventive measures, including maintaining a healthy lifestyle, regular exercise, and adopting ergonomic practices, contribute to overall musculoskeletal well-being [5]. Early detection and appropriate management of these conditions are crucial for minimizing their impact and optimizing long-term health.

### 1.2 The role of conventional treatments and their limitations

Musculoskeletal diseases are chronic conditions that affect the bones, joints, muscles, and connective tissues, leading to pain, inflammation, stiffness, and impaired mobility. Conventional treatments for these conditions mainly aim to alleviate symptoms and improve function, but they have limitations that necessitate a constructive approach to exploring alternative modalities.

Pharmacological interventions, such as nonsteroidal anti-inflammatory drugs (NSAIDs), analgesics, and disease-modifying antirheumatic drugs (DMARDs), are commonly used to manage pain, inflammation, and disease progression [6]. However, they often come with adverse effects, such as gastrointestinal problems, cardiovascular risks, and immune suppression, that limit their long-term use and overall safety [7]. Therefore, patients and healthcare providers must weigh the potential benefits and risks of these medications and monitor their effects regularly.

Physical therapy is another common treatment modality for musculoskeletal diseases, as it can improve mobility, strength, and functionality. However, its success depends on patient compliance and access to specialized care, which may not be feasible for all patients [8]. Moreover, physical therapy may not address the underlying causes of the disease, such as joint degeneration, muscle atrophy, or autoimmune dysfunction.

Invasive procedures, such as joint surgeries or injections, can provide significant relief and restore function in some cases [9,10]. However, they carry surgical risks, such as infection, bleeding, or nerve damage, and may not be suitable for all patients. Moreover, these procedures are usually costly, require hospitalization, and involve a recovery period that may disrupt the patient’s daily activities [11].

Therefore, it is necessary to take a constructive approach and explore innovative therapeutic modalities to address the limitations of conventional treatments. One such modality is electromagnetic field (EMF) therapy, which uses low-frequency electromagnetic waves to stimulate tissue repair, reduce inflammation, and modulate pain signals. EMF therapy is non-invasive, painless, and has minimal side effects, making it a safe and convenient alternative to conventional treatments [12,13]. Moreover, EMF therapy can be used alone or in combination with other therapies, such as physical therapy or pharmacological interventions, to provide personalized and comprehensive care for musculoskeletal diseases.

## 2. Rationale for Electromagnetic Field Stimulation

### 2.1 Historical Perspective

Over thousands of years, the application of EMFs in medical and health contexts traces its roots back to ancient times, with the earliest written records dating to 4,000 BC, describing the use of catfish for therapeutic purposes. Notably, in A.D. 46, Scribonius Largus recommended torpedo fish to alleviate headaches and gouty arthritis, marking an early instance of the medical application of electricity [14].

The systematic study of EMFs in medicine gained momentum in the 18th and 19th centuries when European and American scientists explored electromagnetism’s potential health benefits [15]. In the early 1800s, the relationship between physical forces, including mechanical, electrical, and magnetic forces, and bone biology was recognized. This period also saw key developments like Oersted’s observation of the connection between magnetism and electricity in 1820 and Ampere’s development of the electromagnet [16,17].

In 1832, Faraday’s confirmation of electromagnetic induction laid the groundwork for understanding how electric charges could be transferred [18]. In 1865, James Clerk Maxwell stated the reciprocal relationship between electric and magnetic fields, contributing to the understanding of their interplay. This concept is now integral to the development of electromagnetic devices affecting biological tissues [19].

In the mid-20th century, a study by Fukada and Yasuda on the piezoelectric properties of dry bone, along with subsequent research on the electrical properties of hydrated bone, paved the way for investigating therapeutic applications of EMFs in musculoskeletal disorders (MSDs) [20]. Various technologies, including extracorporeal shockwaves, electrical and electromagnetic stimulations, laser, mechanical, and ultrasound, have been explored for biophysical stimulation of bone formation.

The 1960s and 1970s witnessed advanced research demonstrating that EMFs could stimulate bone growth, enhance blood flow, and reduce inflammation. This led to the acceptance of EMF therapy for musculoskeletal disorders, particularly Pulsed Electromagnetic Field (PEMF) therapy, and magnetotherapy post-World War II [21]. In 1970, Dr. Andrew Bassett and Dr. Arthur Pilla collaborated with Dr. Becker and created a non-invasive PEMF device that succeeded in healing a non-union fracture [22]. The device was called a bone growth stimulator (BGS). These quasi-rectangular and quasi-triangular PEMF signals were approved by the FDA in 1979 for use solely in the United States and were specifically intended for non-union/delayed fractures [23].

PEMF signals with comparable characteristics have been utilized successfully to prevent osteoporosis, even in individuals who have undergone ovariectomy [24]. Research on the use of electricity in medicine persisted into the 20th century, moving beyond bones to explore its potential for evolution, acupuncture, psychic phenomena, and healing. The FDA has approved magneto-therapy for the treatment of various conditions, including fractures, cervical spine surgeries, depression, pain, edema, etc. [25–27]. Ongoing research aims to understand its mechanisms and optimize its application in diverse clinical settings.

### 2.2 Basic principles of electromagnetic field therapy

The electric (E) and magnetic fields (H) are two components of the EMF, which vary in time and move together in space. Both quantities are vectors and have both magnitude and direction. An electric field is generated when an electric charge is present, while a magnetic field is generated when there is a flow of electric charges or an electric current. Electric fields and magnetic fields are strongest near their sources and decrease rapidly in strength with increasing distance from the source. The E field is measured in volts per meter, and the H field is measured in amperes per meter. Magnetic flux density (B) is the other measure of magnetic fields, and it is expressed in Tesla or Gauss [28]. Diagnostic medical imaging equipment such as magnetic resonance imaging (MRI) is typically 1.5–3 T [29].

Magnetotherapy involves six groups of electromagnetic fields [30]: (a) Static magnetic fields (SMF) are non-time varying fields associated with permanent magnets and direct (non-time varying) electric current [31], (b) Low-frequency sine wave electromagnetic fields have been extensively used for treating various types of cancer. The commercially available frequencies of 50 Hz and 60 Hz have been widely recognized as the most effective for this purpose [32], (c) PEMF are characterized by low-frequency fields with specific shapes and amplitudes. The challenge lies in the diverse range of commercially available PEMF devices, making it difficult to compare their physical and engineering attributes, thus hindering the analysis of potential biological and clinical effects [33], (d) Pulsed radiofrequency (PRF) uses specific frequencies within the radiofrequency range for medical and scientific purposes, such as 13.56 and 27.12 [34], (e) Transcranial magnetic or electric stimulation (TMS/TES) involves the application of short yet intense magnetic pulses to target specific areas of the brain [35], and (f) Millimeter waves, with a high-frequency range of 30–100 GHz, have been utilized in the diagnosis of diseases [36].

Static magnetic fields and lower frequency bands are of particular interest in medical applications due to their nonionizing and nonthermal characteristics. Additionally, PEMFs, a therapeutic modality, show promise for treating musculoskeletal disorders, degenerative synovial joints, and cerebrovascular diseases, although the exact mechanisms remain largely unknown. Each group uses different techniques and frequencies, which presents challenges in comparing their biological and clinical effects. EMFs can cause thermal and nonthermal effects, with thermal effects occurring only at frequencies exceeding 10 MHz, which can cause tissue burns [37,38]. Research into the effects of EMFs has revealed both positive and negative outcomes, leading to controversy surrounding their use in treatment and diagnostics [39].

### 2.3 Mechanism of action

EMF exerts its effects on bone and cartilage growth and repair through various mechanisms rooted in fundamental physical concepts. Firstly, the piezoelectric effect, discovered in 1957, elucidates how bone, a crystalline structure, generates an electric potential in response to deformation [40]. This phenomenon underscores the dynamic interplay between mechanical forces and electrical signals in bone tissue. Moreover, the streaming potentials observed in cartilage, documented in 1969, reveal the generation of electric currents concurrent with compression, influencing the behavior of chondrocytes and potentially contributing to cartilage maintenance and repair [41]. Additionally, Wolff’s Law, established in 1892, highlights the pivotal role of mechanical strain in regulating the equilibrium between bone formation and resorption. This principle extends beyond bone tissue, as the benefits of weight-bearing exercise in preserving bone density also apply to cartilage [42].

Regarding the administration of EMF to biological tissues, two primary modalities exist, capacitive coupling and inductive coupling. Capacitive coupling enables the application of PEMF without direct skin contact, albeit requiring electrode placement on the skin in direct capacitive coupling. Conversely, inductive coupling induces currents within the body’s conductive tissues by generating an electric field from the magnetic field, bypassing the need for electrode-skin contact [43].

**Figure 1:**
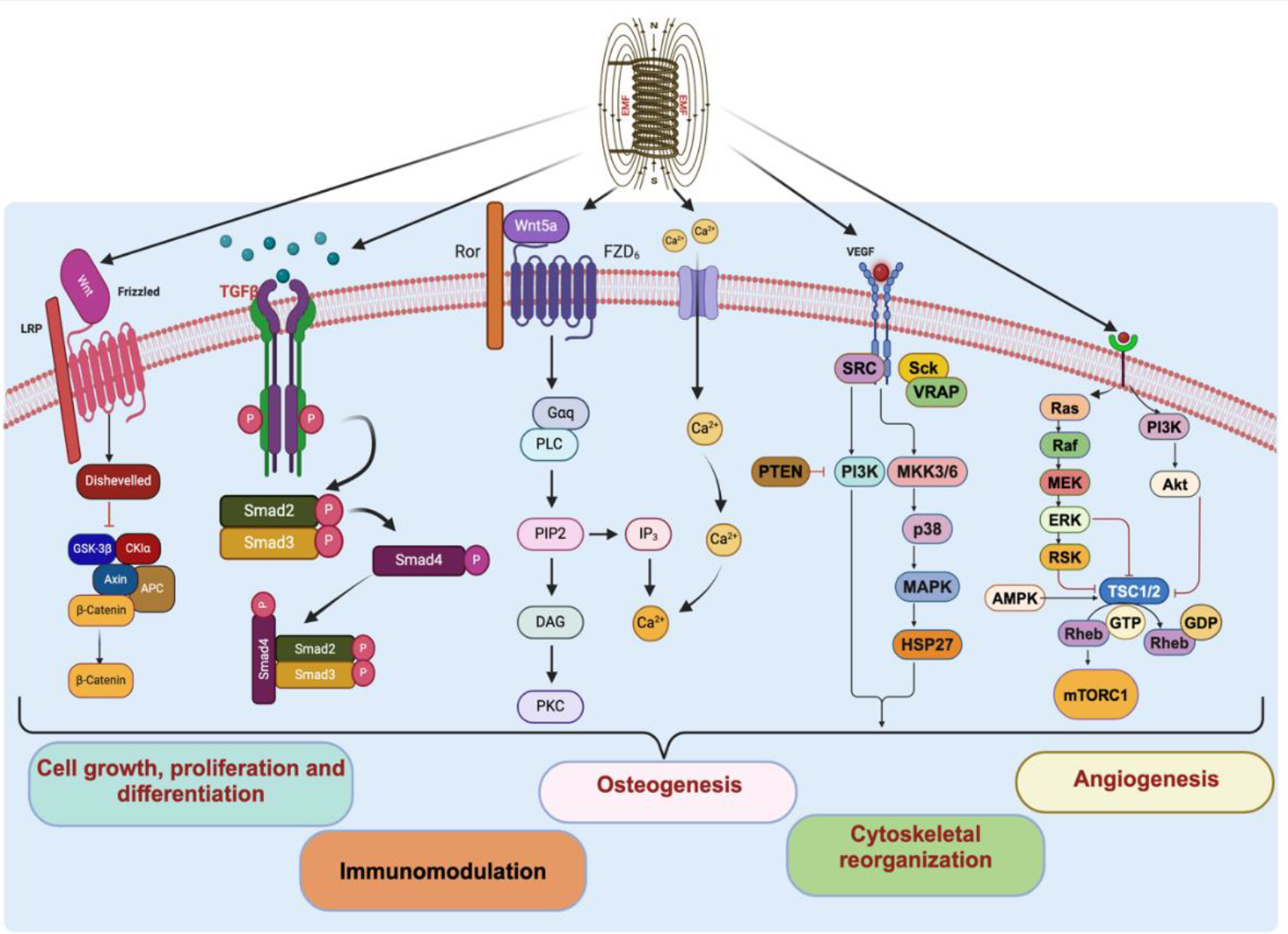
The underlying signaling pathways induced by electromagnetic field (EMF) therapy showing the effect of intracellular molecules in the immunomodulation and cytoskeletal reorganization in the diseased tissue.

Like extracorporeal shock wave therapy (ESW), PEMF serves as a physical stimulus that perturbs the cell membrane, initiating intricate intracellular pathways (Figure 1). This disruption can lead to the formation of lipid “nanopores” in the plasma membrane, facilitating the influx of ions such as Calcium (Ca) from the extracellular environment [44]. Additionally, PEMF may directly affect phospholipids within the plasma membrane, prompting the generation of various second messengers. These messengers play critical roles in activating diverse intracellular signal transduction pathways and triggering the activation of protein kinase C, collectively contributing to the overall cellular response, and ultimately influencing bone and cartilage growth and repair (Figure 1). Overall, electromagnetic field may induce immunomodulatory response in the injured tissue [45].

### 2.4 TGF-β pathway

TGF-βs and BMPs are versatile growth factors that belong to the TGF-β superfamily. When they interact with TGF-β type 1 and type 2 receptors, or BMP serine/threonine kinase receptors, they initiate a signaling cascade through two pathways: the canonical (or Smad-dependent) and non-canonical (or Smad-independent) pathways [46]. Shock wave therapy stands out as an effective and noninvasive approach for managing various tendon pathologies. A recent study delved into the impact of shock waves on tenocyte proliferation and collagen synthesis, shedding light on the underlying biochemical mechanisms. The findings revealed that shock waves can stimulate tenocyte proliferation and collagen synthesis. This stimulation occurs through the early up-regulation of crucial factors like PCNA and TGF-β1, along with increased gene expression of collagen 1 and collagen 3. Furthermore, shock waves prompt the release of endogenous nitric oxide (NO) and the synthesis of TGF-β1 and collagen proteins. This underscores the significance of TGF-β1 in the beneficial outcomes of shock wave therapy, emphasizing its role in promoting tendon healing and regeneration [47]. A study proposed the use of (PEMF) to enhance the chondrogenesis of MSCs for cartilage repair. The study found that the application of PEMF, combined with superparamagnetic iron oxide nanoparticles (SPIO) labeling, could activate the TGF-/SMAD signaling pathways and improve the chondrogenesis of MSCs. Biochemical and gene expression analysis showed upregulation of certain cartilage biomarkers, SOX9 and COL2A1. The expression of TGF-β, p-SMAD2, and p-SMAD2/3 increased in TGF-β treated BMSCs, and cartilage-specific proteins SOX9 and COL2A1 were elevated accordingly. The study suggested that SPIO-PEMF could function as a TGF-β signal to activate intracellular downstream SMADSs and eventually potentiate cartilage-specific markers during the process of BMSC differentiation [48]. Researchers have also found that PEMFs promote osteogenic differentiation and maturation in rat calvarial osteoblasts (ROBs) by activating the BMP-Smad1/5/8 signaling pathway. PEMF treatment upregulates the expression of BMPRII, the primary receptor for BMP ligands, and facilitates its localization at the bases of primary cilia. Disruption of primary cilia formation hinders the PEMF-induced upregulation of BMPRII and its ciliary localization. Knockdown of BMPRII expression attenuates the osteogenic effects of PEMFs, suggesting BMPRII as a crucial link between primary cilia and BMP-Smad1/5/8 signaling. In summary, PEMFs stimulate osteogenic differentiation and maturation through primary cilium-mediated upregulation of BMPRII expression, subsequently activating the BMP Smad1/5/8 pathway [49]. Another study was conducted to investigate the potential of extreme low frequency (ELF)-PEMFs in mitigating the adverse effects of cigarette smoking on bone health. The researchers utilized immortalized human mesenchymal stem cells (SCP-1 cells) impaired by cigarette smoke extract (CSE), exposing them to ELF-PEMFs at 16 Hz for varying durations ranging from 7 to 90 minutes. The results indicated that a 30-minute daily exposure to a specific ELF-PEMF regimen was the most effective in promoting cell viability, enhancing adhesion, and spreading, accelerating migration, and protecting TGF-β signaling from the harmful effects induced by CSE [50].

### 2.5 Ca^2+^ signaling

The conversion of the PEMF signal into a biological signal is heavily dependent on the presence of intracellular Ca^2+^. Research has shown that the PEMF signal can induce the release of calcium ions within cells, which subsequently activates calmodulin in the cytoskeleton [51]. The activation of calmodulin leads to notable changes in various physiological processes such as enhanced cell proliferation, altered signal transduction, and an increase in the synthesis and secretion of growth factors ultimately enhancing the viability of the cell [52]. During osteogenesis, intracellular Ca^2+^ release relies heavily on the voltage-gated Ca channels (VGCCs), particularly the L-type [53]. Research has found that exposure to PEMF can increase the expression of VGCCs in MSCs, ultimately enhancing osteogenesis [54]. Furthermore, the PEMF stimulation can lead to higher levels of nitric oxide, which, in turn, leads to increased cGMP synthesis and protein kinase G activation. Through the Ca^2+^/nitric oxide/cGMP/protein kinase G pathway, this cascade can promote osteoblast differentiation and maturation, as well as bone repair [55]. Lastly, various studies have reported the interplay between Ca^2+^, ERK, PKA, and PKG signaling under PEMF stimulation, which ultimately leads to the therapeutic effect of PEMFs on bone repair and reduced pain in patients by modulating the release of inflammatory cytokines [56–59].

Exposure to PEMFs stimulates the movement of MSCs in a manner that relies on calcium inside the cell. PEMFs increase the level of calcium within the cell, which in turn activates focal adhesion kinase (FAK) signaling. This leads to an increase in the activity of Rho GTPase and the formation of a more extensive F-actin network. As a result, the cytoskeleton undergoes reorganization, and the cells move [60]. Another in vitro assessment was conducted to evaluate the impact of extracorporeal shock wave (ESW) intensity on bone marrow mesenchymal stem cells (BMSCs). The outcomes of this evaluation revealed that the activation of Wnt5a/Ca2+ signaling was observed, which led to significant changes in the expression levels of associated genes and proteins, such as Wnt5a, PKC, PLC, and CaMKII. Additionally, the results revealed that ESW prevented histological changes in osteoarthritis (OA) [61].

### 2.6 MAPK pathway

The MAPK pathway is a signaling system found in different organisms that regulates cellular responses. It transduces signals to different cellular compartments, regulating cell proliferation, differentiation, migration, and death [62]. PEMF was shown to treat motor system diseases, particularly bone, joint, and tendon injuries. It induced extensive biological effects, including increased cell proliferation. An in vitro study showed that low-frequency PEMF enhanced the proliferation of mouse skeletal myoblasts via activation of the MAPK/ERK pathway. Exposure of C2C12 myoblasts to PEMF increased the phosphorylation level of ERK, while p38 MAPK and JNK pathways remained unaffected. Pretreatment of the cells with the MEK1/2 inhibitor inhibited C2C12 cell proliferation. These results suggest that PEMF could provide a promising therapeutic approach for enhancing myoblast proliferation through MAPK/ERK pathway activation [63]. Another study used porous scaffolds made of polycaprolactone (PCL) and nano hydroxyapatite (nHA) as cell carriers for BMSCs. The BMSCs were treated with EMF. It was found that BMSCs stimulated by EMF possess splendid osteogenic capability. The scaffold loaded with BMSCs stimulated by EMF also accelerated intervertebral fusion successfully. Mechanistically, EMF regulates BMSCs via BMP/Smad and MAPK-associated p38 signaling pathways [64]. ELF-PEMF treatment enhanced protein content, mitochondrial activity, ALP activity, and promoted mineralized matrix formation in osteoblasts. The positive effects were mediated through the activation of the ERK1/2 signaling pathway, which was observed in our experiments with primary human osteoblasts treated with ELF-PEMF [16]. Inhibition of ERK1/2 signaling with U0126 prevented activation of AP activity and matrix mineralization by ELF-PEMF treatment [16]. Therefore, ERK1/2 signaling was pivotal for the observed positive effects of ELF-PEMF treatment on osteoblast function [65]. A recent study demonstrated that the use of a magnetofection system to deliver miR-21 into BMSCs and human umbilical vein endothelial cells (HUVECs) resulted in increased osteogenesis and angiogenesis in vitro and in vivo. The study also found that the co-stimulation of EMF and iron oxide nanoparticles (IONPs) was found to enhance magnetofection efficiency and promote osteogenesis and angiogenesis through the p38 MAPK pathway as evidenced by increased protein expression levels of phosphorylated p38, tau, and HSP27 (p-p38, p-tau, and p-HSP27, respectively). This approach could potentially be used as a therapeutic intervention for various orthopedic diseases, including intervertebral fusion procedures [66].

### 2.7 Wnt/β-catenin signaling

The extracellular Wnt ligands bind to their seven-pass transmembrane Frizzled receptors and co-receptors of the arrow/Lrp family, such as LRP5 and LRP6, simultaneously to initiate the canonical Wnt/β-catenin signaling pathway. This process leads to the stabilization of β-catenin in the cytoplasm, facilitating its translocation to the nucleus, where it interacts with transcription factors to regulate gene expression [67]. The Wnt/β-catenin signaling pathway plays a crucial role in PEMF-induced osteogenic and chondrogenic differentiation of mesenchymal progenitor cells, bone formation, and repair. Studies have shown that PEMF increased gene and protein expressions of Wnt3a, β-catenin, and OPG in tibial subchondral bone of knee OA rats, promoting the activation of Wnt/β-catenin signaling and OPG/RANKL/RANK signaling. PEMF may help preserve the subchondral bone’s structural integrity in knee OA [68]. A study conducted in an in vitro environment found that subjecting mesenchymal stem cells to single-pulsed electromagnetic field (SPEMF) treatment for 3 min daily can enhance their ability to differentiate into osteogenic cells and accelerate bone growth. This is achieved through the activation of the Wnt signaling pathway, which is confirmed by the increased gene expression of Wnt1, Wnt3a, Wnt10b, Fzd9, ALP, and Bmp2 [69]. Recent findings suggested that Wnt signaling also mediated diabetic bone deterioration. The study aimed to understand how PEMF regulated bone quality and metabolism from the perspective of Wnt signaling. The study provided strong evidence that PEMF up-regulated Wnt3a but not Wnt1 or Wnt5a, and stimulated the expression of β-catenin and p-GSK-3β proteins in mandibular osteoblasts from diabetic mice [70]. Another study aimed to evaluate the effect of PEMF on subchondral bone microstructure through the Wnt/β-catenin signaling-associated pathway in rats with knee osteoarthritis (OA) induced by low-dose monosodium iodoacetate (MIA). The results showed that PEMF treatment upregulated tibial subchondral bone gene expressions including Wnt3a, b-catenin, OPG, and OPG/RANKL, which were down-regulated in low-dose MIA rats. Thus, modulation of PEMF in subchondral bone metabolism and structure in low-dose rats might be associated with activation of canonical Wnt signaling and OPG/RANKL/RANK signaling [68].

Results from an in vivo assay study showed that PEMFs could effectively reverse bone mass loss and deterioration of bone microarchitecture in hind limb-suspended ovariectomized rats. This was analyzed by micro-CT and evaluated by a three-point bending test, suggesting that activating the Wnt/Lrp5/β-catenin signal pathway through PEMF exposure was beneficial for bone disorders. PEMF exposure significantly promoted the overall gene expressions of Wnt1, LRP5, and β-catenin in the canonical Wnt signaling, without any noticeable impact on either RANKL or RANK gene expressions [71]. PEMF exposure was studied for its effect on healing delayed union femur fractures in rats. The PEMF group was exposed for 4 hours daily for 5, 10, 18, and 28 days. Histological and RT-PCR examination showed higher gene expression of Wnt10b, Wnt5a, and β-catenin in the PEMF group compared to the control group. The PEMF group had less fibrous tissue in the fracture gap and significantly increased alkaline phosphatase activity on day 10. It was concluded that PEMF exposure can speed up delayed union fracture healing through the Wnt signal pathway [72].

### 2.8 Other pathways

The Notch signaling pathway, known for its high conservation, plays a critical role in governing cell fate decisions and skeletal development. In an in vitro study, BMSCs were cultured in an osteogenic medium, and PEMFs were applied. Researchers found that PEMFs increased osteogenic markers and activated the Notch pathway, specifically Notch4, Dll4, Hey1, Hes1, and Hes5 genes. Inhibiting the Notch pathway led to significant inhibition of osteogenic markers and Notch target genes, indicating that the Notch pathway plays a crucial role in PEMF-stimulated osteogenic differentiation. These findings may contribute to improving autologous cell-based bone defect regeneration in orthopedics by understanding the role of Notch signaling in PEMF-induced osteogenesis [73].

Mouse genetic studies have shown that mTOR pathways play a crucial role in regulating skeletal development and homeostasis [74]. A recent study found that a composite scaffold, combining Hydroxyapatite-Collagen type-I (HAC) and PLGA-PEG-PLGA thermogel with EMF stimulation, significantly improved the repair of osteochondral defects in rabbits. In vitro experiments showed that EMF treatment promoted BMSC proliferation and chondrogenic differentiation, partly through activation of PI3K/AKT/mTOR and Wnt1/LRP6/β-catenin signaling pathways [75].

## 3. Application of EMF Therapy in Musculoskeletal Disorders

**Figure 2:**
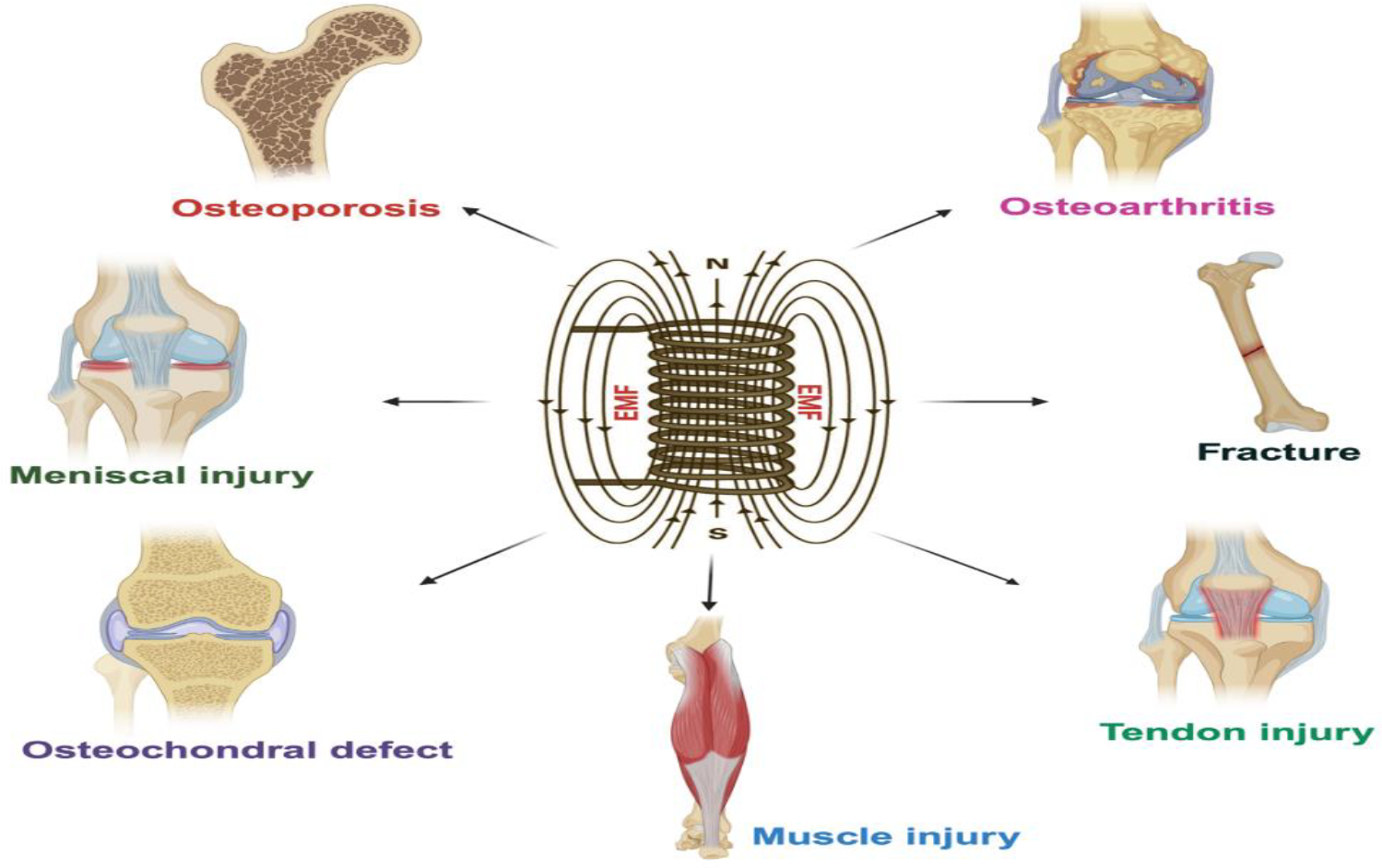
Therapeutic application of EMF in musculoskeletal disorders.

Electromagnetic fields (EMFs) have been investigated for various applications in musculoskeletal medicine, primarily in the context of therapeutic interventions (Figure 2).

## 4. Bone

### 4.1 Osteoporosis

Osteoporosis is a condition of the skeletal system in which the bone mineral density (BMD) is low, and the bone architecture is disrupted. This leads to an increased risk of bone fragility, which is commonly observed in postmenopausal women and can be a costly condition [76]. Osteoporosis is a significant clinical problem that can cause pain and increase the risk of fractures [77]. Various treatments are available for osteoporosis; however, the use of these treatments is limited by their multiple side effects, high cost, and low persistence [78]. Electromagnetic field (EMF) therapies have gained popularity in recent decades as a safe, effective, and noninvasive treatment option for osteoporosis [79].

A study was conducted on rats with osteoporosis using pulsed electromagnetic fields (PEMF) for 40 minutes per day, while the control group was treated with alendronate. The outcome showed that the bone structural mechanical index and maximum stress of the right femur in the alendronate group were significantly increased after 8 weeks compared to the control group. However, only the maximum stress and strain were found to be improved in the same group after 12 weeks. The serum osteocalcin (BGP) and bone morphogenetic protein-2 (BMP-2) concentrations in the PEMF and alendronate groups were increased after 2 weeks, but this increase was not synchronized. After 8 weeks, the BGP and BMP-2 levels in the PEMF group were noticeably elevated compared to the alendronate group. The findings suggest that PEMF can effectively improve the mechanical stability of bone structure more gently and sustainably than alendronate [80].

Another study investigated the effectiveness of combining PEMF stimulation and sclerostin monoclonal antibody (Scl-Ab) in the treatment of osteoporosis. The experiment was conducted on a rabbit model of postmenopausal osteoporosis, and specimens were fixed with pedicle screws in the L4 vertebral body. After eight weeks of treatment, the results showed that the combination of PEMF and Scl-Ab therapies significantly increased bone mineral density (BMD) by 35.0% compared to single therapies. Furthermore, the maximum pulling force of pedicle screws increased by 19.1%, and the maximum failure power consumption of pedicle screws increased by 33.6% in the combination therapy group. These findings suggest that the combination of PEMF and Scl-Ab therapies could have significant clinical potential [81].

A recent study aimed to compare the effect of two different modalities of pulsed electromagnetic field (PEMF) therapy with pharmacological treatment on ovariectomized osteoporosis in rats. The results showed that exposure to PEMF at 40Hz significantly reduced osteoporotic bone loss, while PEMF at 25Hz led to further progression of osteoporosis. PEMF at 40Hz was found to be more effective than pamidronate, vitamin D, and calcium supplementation in restoring osteoporosis and attenuating bone fragility [82].

### 4.2 Osteoarthritis

Osteoarthritis (OA) is a degenerative disease that affects one or more joints, causing pain, swelling, deformity, instability, or impaired joint function [83]. Knee OA is the most prevalent form of OA, accounting for 85% of the worldwide OA burden [84]. A multitude of conservative treatment options is available, including physiotherapy, TENS, acupuncture, local heat, and cold application, as well as pharmacological analgesia with NSAID [85]. PEMF therapy, an emerging modality for the treatment of musculoskeletal disorders, has been approved by the American FDA and has a broad range of indications for use [86].

A study was conducted to investigate how PEMF affected osteoarthritis (OA) in mice. The mice underwent destabilization of the medial meniscus (DMM) surgery and were treated with PEMF or a sham PEMF for 1 hour per day for a total of 4 weeks. The results of the study showed that PEMF had a positive effect on reducing pain, cartilage degeneration, synovitis, and trabecular bone microarchitecture in wild-type (WT) mice. However, these effects were reduced in mice that lacked IL-6 or TNF-α. PEMF also reduced the expression of IL-6 and TNF-α in cartilage and improved cartilage matrix, chondrocyte apoptosis, and autophagy. The study concluded that PEMF could delay the progression of OA by inhibiting TNF-α and IL-6 signaling [87].

A recent study has introduced a new production system for small extracellular vesicles (sEVs) that can improve their therapeutic properties for treating osteoarthritis (OA). The system stimulates MSCs using electromagnetic field (EMF) and ultrasmall superparamagnetic iron oxide (USPIO) particles. The resulting EMF-USPIO-sEVs activate anabolic pathways, inhibit catabolic activities, promote M2 macrophage polarization, and transport decreased miR-99b-5p levels into recipient cells. In an OA mouse model, EMF-USPIO-sEVs reduce OA severity, augment matrix synthesis, and decelerate OA progression through the microRNA-99b/MFG-E8/NF-κB signaling axis. This study highlights the therapeutic potential of EMF-USPIO-sEVs in re-establishing chondrocyte homeostasis and promoting M2 macrophage polarization for OA treatment [88].

Recently, another study aimed to observe the effect of pulsed electromagnetic field (PEMF) on the degeneration of knee joint cartilage in aged rats. The results showed that PEMF improved osteoarthritis in aged rats by inhibiting chondrocyte senescence, alleviating articular cartilage degradation, and inhibiting subchondral bone osteoporosis by suppressing the expression of P53/P21. The study also found that PEMF treatment increased the bone volume fraction, bone mineral density, and number of trabeculae while decreasing the trabecular separation in the tibia of rats in the PEMF group compared to the aged group [89].

### 4.3 Bone fracture

Bone fractures are becoming a critical issue for public health, especially as the world’s population ages. Nonunion, which is a complication resulting in delayed or non-healing of fractures, affects many people [90]. Nonunion can be exacerbated by systemic risk factors such as smoking, diabetes, and cachexia, as well as local factors such as poor vascularity and inadequate fixation [91]. While nonunion is currently treated with surgery, there is a growing need for non-invasive therapies that can speed up the healing process. Electromagnetic (EM) field stimulation is a promising therapy that can help improve bone healing.

A study on mice aimed to explore the potential of low-intensity EM field stimulation for bone fracture repair. The results revealed a significant increase in osteogenic differentiation in vitro, accompanied by an increase in mitochondrial membrane potential and respiratory complex I activity, following exposure to an EM field of 10 Gauss for four days. Moreover, in vivo experiments demonstrated that EM field stimulation led to improved biomechanical properties and increased callus bone mineralization, indicating enhanced fracture repair. The findings of this study suggest that EM field therapy could be a promising intervention for bone fracture repair by activating mitochondrial OxPhos [92].

In a study involving 56 male Sprague Dawley rats, aged 3–4 months and in good health, an assessment was done on the healing of delayed union fractures. There were no infections or implant protrusions. The study was carried out in four phases from the second to the fifth week. The rats were exposed to an Extreme Low Frequency-Pulsed Electromagnetic Field (ELF-PEF), and it was found that bone fracture healing happened faster than the control group. In the follow-up test, significant differences in RUST radiology scores were observed each week. The study concluded that exposure to ELF-PEF accelerated the healing of bone fractures since the second week of exposure [93].

### 4.4 Bone loss

Radiotherapy is a common cancer treatment that can cause bone damage, including reduced bone mass and fragility [94]. This happens because radiation suppresses bone-forming cells called osteoblasts, inhibiting bone formation [95]. EMFs could be a potential remedy, as they stimulate osteoblast growth.

A study reports a non-invasive technique based on a noninvasive EMF that inhibits radiotherapy-induced bone loss. The PEMF at 15 Hz and 2 mT induces notable Ca2+ oscillations depending on interactions between ciliary polycystins-1/2 and endoplasmic reticulum, which activates the Ras/MAPK/AP-1 axis and subsequent DNA repair Ku70 transcription. PEMF promotes the specific activation of the molecular expression of the Ras/MAPK pathway. The study also established osteoblast specific Ku70 knockout mice and found that these mice were more vulnerable to ion radiation and resistant to PEMF treatment. The results provide strong evidence for the therapeutic potential of PEMF as a noninvasive approach against radiotherapy-induced bone loss [96].

### 4.5 Tendon

Tendinopathy is a condition that causes pain and reduced function due to abnormalities in damaged and diseased tendons. Overuse tendinopathies are most common and affect tendons in different parts of the body, such as the rotator cuff tendon, medial and lateral elbow epicondyles, patellar tendon, gluteal tendons, and the Achilles tendon [97]. It can be classified as a failure in the homeostatic response of the tendon and is mainly seen in active workplaces and sports fields. It can lead to integrant morbidity and disability [98]. Conventional treatments include NSAIDs and corticosteroid injections, but their long-term benefits are still being debated [99]. Other adjuvant therapies such as rehabilitation exercises, low-level laser therapy, and shock wave therapy are also used [100–102]. Tendons are mainly made of collagen, and tenocytes are responsible for maintaining healthy tendons [103]. Inadequate collagen synthesis and matrix degradation cause tendinopathy. EMFs have shown prospective effects on tendon disorders in vivo and in vitro.

In a study evaluating the role of PEMFs in improving the tendon healing process, a total of 68 Sprague Dawley rats received a single injection of type I collagenase in Achilles tendons to induce tendinopathy. Daily exposure to PEMFs (1.5 mT and 75 Hz) for up to 14 days was found to improve the fiber organization, decrease cell density, vascularity, and fat deposition, and restore the physiological cell morphology compared to untreated tendons. The most effective protocol was found to be PEMF exposure for 14 days during the mid-acute phase of the pathology (7 days after induction). These findings suggest that PEMFs represent a promising conservative treatment for tendinopathy, although further investigations regarding clinical evaluation are needed [104].

Another study aimed to compare the effectiveness of topical dimethyl sulfoxide (DMSO) with a combination of topical DMSO and PEMF for the treatment of equine superficial digital flexor (SDF) tendonitis. The study involved two groups of polo ponies, with the control group receiving DMSO and controlled exercise, and the experimental group receiving the same protocol plus PEMF on the injured tendon. The study found that while there was a slight improvement in fiber alignment and echogenicity in the test group, there was no significant impact on clinical evaluation. The study suggested that more extended application at different frequencies may be necessary to elicit a favorable outcome of PEMF for the treatment of SDF tendonitis [105].

A study on postoperative rotator cuff (RC) healing in rats, using PEMF therapy, showed that focused PEMF treatment improved biomechanical elasticity parameters and collagen organization. The study involved 30 rats that underwent acute bilateral supraspinatus tear and repair, using a miniaturized electromagnetic device (MED) implanted on the right shoulder. The results suggest that PEMF generated by the MED may enhance early postoperative tendon-to-bone healing in acute rat supraspinatus detachment and repair models [86].

In an in vitro assessment, it was observed that a single treatment of Rat primary tenocytes with single-pulsed electromagnetic fields (SPEMF) at a frequency of 0.2 Hz demonstrated an up-regulation in the expression of tenogenic genes (Col1a1, Col3a1, Scx, Dcn). Concurrently, there was a notable down-regulation in the expression of the inflammatory gene MMP1. Furthermore, following five days of SPEMF stimulation (3 minutes per day), there was a significant increase in protein levels associated with collagen type I and total collagen synthesis. These findings suggest that SPEMF has the potential to mitigate the imbalance between matrix synthesis and degeneration observed in tendinopathy. Consequently, SPEMF may emerge as a promising strategy for therapeutic intervention in tendon disorders [106].

In another in vitro study, PEMFs were found to effectively reduce inflammation and promote the synthesis of tendon markers in human tendon cells (TCs), suggesting their potential as a therapeutic intervention for the treatment of tendon injuries and inflammation. The study demonstrated that PEMFs exerted a notable modulation on TCs, promoting the upregulation of COL3A1 and IL-33 secretion. In the presence of IL-1β, TCs exhibited an upregulation of ADORA2A, SCX, and COL3A1 expression, and an increase in IL-6, IL-8, PGE2, and VEGF secretion. Impressively, after exposure to PEMFs and IL-1β, IL-33 was upregulated, while IL-6, PGE2, and ADORA2A were downregulated, further underscoring the potential of PEMFs as a therapeutic intervention [107].

### 4.6 Muscle

An in vitro study conducted on skeletal muscle cells has shown that complex magnetic fields can be used to control intracellular signaling in these cells. The fields induce a temporary depolarization of cellular membranes, leading to ion influxes and biochemical reactions that activate RyR and promote actin polymerization. The observed increase in cytosolic calcium is related to the emergence of eddy currents induced by moderate-strength alt-magnetic fields with short exposures. This study provides a universal framework for triggering intracellular Ca^2+^ signaling using alternating magnetic fields, and it opens up possibilities for developing new clinical devices to treat myopathies that are linked to defective calcium regulation in muscle cells [108].

In a recent study conducted in vitro, researchers aimed to evaluate the potential of PEMF to stimulate the early regeneration of human skeletal muscle cells (SkMC). The study revealed that 1.5 mT PEMF can promote SkMC proliferation without causing cell apoptosis or significant impairment of metabolic activity. Furthermore, the same PEMF treatment can accelerate the regenerative process by inducing cell migration to close wounds. The study also found that PEMF sustains the expression of antioxidant enzymes, such as HSP70, thioredoxin, paraoxonase, and SOD2, which can aid in skeletal muscle regeneration following an injury. These findings suggest that PEMF has the potential to increase SkMC regeneration and control inflammatory and oxidative processes following muscle damage [109].

In a comprehensive in vivo study, the long-term impact of chronic exposure to extremely low-frequency electromagnetic fields (ELF-EMF) on the diaphragm muscle in rats was systematically investigated. Twenty-nine newly weaned Wistar Albino rats were exposed to a 50 Hz frequency and 1.5 mT magnetic flux density for 4 hours daily over 7 months. Evaluation encompassed electrophysiological, histological, and biochemical aspects. The results revealed that ELF-EMF exposure did not significantly affect the histological structure or mechanical activity of the diaphragm muscle. Most muscle bioelectrical activity parameters remained unchanged, with minimal alterations observed. Biochemical analyses, including blood serum ion levels and enzyme-specific activities in muscle tissue, showed no significant deviations, indicating relative stability. While some small changes in bioelectrical activity parameters were noted, their clinical relevance appeared limited. Overall, chronic exposure to ELF-EMF exhibited no substantial adverse effects on the diaphragm muscle in rats under the specified experimental conditions [110].

Another in vivo experiment was carried out to assess the effects of High-Intensity Focused Electromagnetic (HIFEM) treatment on the structure of porcine muscle tissue. In this study, three Yorkshire pigs received four 30-minute HIFEM treatments, and biopsy specimens were collected from the treatment site. Histologic analysis showed that 2 weeks posttreatment, the muscle mass density increased by 20.56%, the average change in the number of muscle fibers increased by 8.0%, and the mean size of an individual muscle fiber increased by 12.15%. Control samples did not show any significant change in fiber density or hyperplasia. These results suggest that HIFEM could be used for non-invasive induction of muscle growth [111].

A recent in vivo study was conducted to investigate the effects of radiofrequency radiation (RFR) on bone biomechanics and skeletal muscle tissues of diabetic and healthy rats. The rats were exposed to 3.5 GHz RFR for 2 hours per day for 30 days. The study found that exposure to RFR had negative effects on bone biomechanics, including decreased elasticity coefficient and Young’s modulus, increased maximum displacement, and decreased maximum force. Additionally, the study found that diabetic rats experienced greater alterations in oxidative stress parameters than their healthy counterparts. Based on these findings, it is concluded that exposure to 3.5 GHz RFR may have the potential to negatively impact bone quality and structural integrity, especially in diabetic rats [112].

### 4.7 Cartilage

Cartilage injury is damage to the smooth tissue that cushions joints. There are three types of cartilage in the body: elastic cartilage, fibrocartilage, and hyaline/articular cartilage [113]. Cartilage injuries can be caused by sports, falls, repetitive movements, overuse, being overweight, aging, and genetic factors [114]. Cartilage repair and restoration surgeries are limited by factors such as graft availability, donor site morbidity, and difficulty in matching size and surface contours [115]. Engineered cartilage technologies are progressing through the clinical pipeline, but their wider adoption is hindered by economic factors and difficulty in recapitulating native properties. Various in vitro and in vivo studies have demonstrated that PEMFs can be a safe and cost-effective method to aid in cartilage repair.

### 4.8 Articular cartilage

Articular cartilage injuries are a common source of joint pain and dysfunction, and the intrinsic healing capacity for self-repair is poor. However, electrotherapeutic strategies such as PEMFs and applied direct current (DC) electric fields (EFs) via galvanotaxis can promote cartilage healing via cell-mediated repair. PEMF stimulation can promote bovine fibroblast-like synoviocytes (FLS) migration in vitro by 24 h, suggesting that EF stimulation can promote FLS movement. Galvanotaxis DC EF stimulation can help FLS migration within a collagen hydrogel matrix, exhibiting increased incremental and overall speeds of movement. PEMF stimulation can further modulate FLS migration into the bovine cartilage defect region, resulting in elevated GAG and collagen levels following PEMF treatment. Electrotherapeutic stimulation can promote intrinsic cartilage repair via FLS modulation, enhancing direct homing of resident FLS and expediting the rate of cartilage repair without surgical interventions [116].

Another in vitro study was conducted to investigate the effects of ultra-low complex electromagnetic fields on an in vitro cartilage regeneration model. The study found that Limfa^®^ Therapy, delivered by an innovative medical device, was able to induce the modulation of genetic chondrogenesis markers in adipose mesenchymal stem cells (ADSCs) and promote ADSC differentiation when coupled with biochemical stimuli contained in a pro-chondrogenic medium. Limfa^®^ Therapy was also found to have preferred promoting hyaline cartilage formation instead of bone tissue. The findings suggested that Limfa^®^ Therapy could be useful in the clinical treatment of osteoarthritis and could potentially be improved by adding an autologous ADSCs intra-articular injection to boost cell regeneration capacity [117].

A study was conducted to evaluate a composite scaffold made of Hydroxyapatite-Collagen type-I (HAC) and PLGA-PEG-PLGA thermogel, which was stimulated with an EMF to repair cartilage damage. Since regenerative tissue quality is often poor, a safe and non-invasive magnetic therapy was combined with tissue engineering to develop a promising approach for complete cartilage repair. The study utilized bone marrow mesenchymal stem cells (BMSC), which were encapsulated in the thermogel, and then stimulated with EMF to enhance their proliferation and chondrogenic differentiation potential. The study demonstrated that the EMF treatment promoted the activation of the PI3K/AKT/mTOR and Wnt1/LRP6/β-catenin signaling pathways, leading to an increase in the proliferation and chondrogenic differentiation of BMSCs. In vivo, experiments were conducted on rabbits with 4mm femoral condyle defects, and the results confirmed that the scaffold with EMF treatment significantly improved the repair of osteochondral defects, particularly cartilage repair [75].

Literature showed the development of a new type of magnetic gelatin/β-CD/Fe3O4 hydrogel that had good mechanical properties, high biocompatibility, and hydrophilicity. The magnetic hydrogel combined with pulse electromagnetic fields (PEMFs) could effectively repair defective articular cartilage. The combination of magnetic hydrogel and PEMFs promoted the differentiation of bone marrow mesenchymal stem cells (BMSCs) into cartilage in vitro, leading to an increase in the index of cartilage differentiation. The magnetic nano-hydrogel material exhibited a superparamagnetic effect and was co-cultured with the magnetic hydrogel under the stimulation of a pulsed electromagnetic field. The results of the in vivo experiments showed that the magnetic hydrogel combined with BMSCs and PEMFs had a strong repair effect on knee joint injury in rabbits. The study successfully combined tissue engineering and the PEMF approach to repair defective articular cartilage, which could be adaptable in the future for human cartilage tissue engineering treatment [118].

Another type of supramolecular hydrogel, Alg-DA/Ac-β-CD/gelatin hydrogel, was evaluated in combination with PEMF for repairing cartilage. This hydrogel was deemed adaptable to complex clinical situations and had a pre-gel state that allowed for multiple administration routes. The results of the study indicated that using PEMFs in combination with this hydrogel had positive effects on rBMSCs chondrogenic and hypertrophic gene expression both in vitro and in vivo. In vitro, PEMF was found to upregulate the expression of chondrogenic mRNA and downregulate the expression level of RUNX2. In vivo, PEMF enhanced the treatment of rBMSCs-laden hydrogels (P-MSCs + PEMF) on rat osteochondral defect models, leading to increased ECM deposition and higher Young’s modulus and ultimate strength compared to the control group. The study also identified the TNF-α signaling pathway as a potential target pathway in PEMF treatment. Inhibition of ERK and p38 led to changes in the expression level of chondrogenic and hypertrophic markers during MSC’s chondrogenic differentiation [119].

### 4.9 Meniscus

Meniscal tears are common and can lead to long-term disability and osteoarthritis. While arthroscopic surgery is the main treatment, it is not always successful. Partial meniscectomy can worsen the condition [120]. Some biological treatments have been tried, but the results are mixed. Physical therapy, such as shockwave therapy and therapeutic ultrasound, has shown promise in enhancing meniscal healing.

The study aimed to evaluate the effectiveness of PEMF treatment on the healing of meniscal injuries in Sprague-Dawley rats. Macroscopic evaluation showed that the treatment groups, classic signal PEMF (Gclassic), and treatment with the high–slew rate (HSR) signal PEMF (GHSR), exhibited superior healing scores compared to the control group, Gcon. The histological assessment of menisci stained with safranin O/fast green revealed that the defect was filled with dense fibrocartilaginous tissue embedded with round meniscus-like cells in GHSR. In contrast, Gcon exhibited degenerative changes, while Gclassic showed a loosely packed tissue containing a clump of fusiform cells. Immunohistochemical staining of Col-II showed a significant expression of Col-II in the regenerated matrix within the injury site in GHSR, indicating the formation of fibrocartilaginous tissue. Additionally, the Osteoarthritis Research Society International (OARSI) scoring system was used to evaluate the degree of articular cartilage degeneration. The results displayed the highest degeneration score in the control group, Gcon. Lastly, synovitis score analysis revealed significantly more severe synovitis in Gcon and Gclassic than in GHSR [121].

### 4.10 Clinical studies

A comprehensive meta-analysis analyzed 19 Randomized Controlled Trials comprising 1303 women and yielded promising results. The combination of PEMF with conventional medications was found to significantly improve BMD, serum BSAP, ALP, and osteocalcin levels when compared to conventional medications alone. Furthermore, the study confirmed the analgesic effect of PEMF. These results strongly suggest that PEMF may serve as an effective complementary therapy for postmenopausal osteoporosis [122].

PEMF therapy is a useful treatment for relieving knee OA symptoms in the short term, according to a study that analyzed 13 Randomized controlled trials involving 914 unique patients. However, PEMF therapy was not better than other conservative therapies such as physiotherapy. The study identified that the type of control and time of follow-up are two main factors that affect the outcomes of PEMF therapy. PEMFs were found to be more effective than placebo in the short term, as measured by self-reported pain and activity scores. The effect of PEMFs on pain decreases progressively over longer follow-up periods, indicating that the improvements are more likely related to temporary pain and inflammation reduction rather than the direct restoration of cartilage tissue. Also, the study found that the lack of specific protocols for PEMF application has a negligible effect on short-term results in the treatment of OA symptoms [123].

A prospective randomized study was conducted on 40 patients diagnosed with supraspinatus tear. The objective of the study was to compare the effectiveness of transcutaneous electrical nerve stimulation (TENS), ultrasound (US), and PEMF in combination with TENS and US therapy alone. The patients were randomly divided into two groups: PEMF (n=20) and Sham (n=20) groups. The results indicated that there was no significant difference between the PEMF and Sham groups in terms of the Numerical Rating Scale (NRS), University of California–Los Angeles (UCLA) Shoulder Scale, and Shoulder Pain and Disability Index (SPADI) scores. Therefore, it was concluded that the addition of PEMF therapy to the conventional treatment of symptomatic supraspinatus tear would not provide any additional benefit [124].

Another prospective, randomized, single-blind, pre–post-test, controlled experiment aimed to assess the effects of pulsed magnetic field therapy on hand function, grip, and pinch grip strength in male patients with flexor tendon repair. The participants were randomly divided into two groups, one receiving both therapy and exercise, while the other receiving only exercise. After the treatment, the study results showed a significant improvement in the strength of the pinch grip, Michigan Hand Outcomes Questionnaire (MHOQ) ADL, pain, and satisfaction across the groups. However, there was no significant difference in hand grip strength or hand function between the two groups before and after the treatment. The study concluded that pulsed magnetic field therapy is effective in improving physical therapy treatment and increasing the strength of hand and pinch grip in patients after flexor tendon repair in zone II [125].

Another study evaluated the efficacy of PEMF therapy in patients with chronic non-specific neck pain in conjunction with conventional physical therapy. The study was double-blind, prospective, randomized, and placebo-controlled. The participants were divided into two groups - the PEMF therapy group and the control group. Both groups received conventional physical therapy, but only the PEMF group received 20 minutes of actual PEMF therapy while the control group received 20 minutes of sham PEMF. The results showed significant improvement in the visual analog scale (VAS), Neck Pain Disability Scale (NPDS), Short Form-36 (SF-36), and Physician Global Assessment (PGA) after treatment in both groups. However, the PEMF therapy group was not found to be superior to the sham group in terms of improvements in the outcome parameters. Therefore, the study concluded that while PEMF therapy is safe for chronic, non-specific neck pain patients, it does not provide any additional benefit when applied in conjunction with conventional physical therapy [126].

A prospective randomized controlled study was conducted to compare the effectiveness of two types of therapies, interference current (IFC) and PEMF, combined with conventional physical therapy on patients with mechanical chronic low back pain (CLBP). The study involved 40 CLBP patients who were divided into two groups. Group I received a hot pack, ultrasound (US), and IFC combination therapy, while group II received a hot pack, US, and PEMF combination therapy. The study results showed that both PEMF and IFC therapies, in addition to conventional physical therapy programs, were effective in treating mechanical CLBP in terms of pain relief, functional status improvement, and quality of life enhancement [127].

### 4.11 Limitations of PEMF therapy

EMFs have shown potential as a non-invasive treatment option. However, their therapeutic effectiveness is hindered by a lack of understanding of the influence of various parameters, such as frequency, amplitude, duration, tissue type, and field strength, on their biological effects. The variability in EMF response poses challenges in establishing definitive guidelines for their clinical application, as existing studies exhibit diverse treatment protocols with variations in EMF parameters, leading to inconsistency and hindering the formulation of standardized treatment guidelines.

Moreover, individual patient responses to EMFs are influenced by factors such as age, sex, and comorbidities, necessitating personalized research to predict treatment outcomes accurately. The absence of clear guidelines further complicates the implementation of EMF therapy in clinical practice, increasing uncertainty for both patients and medical institutions.

Addressing these challenges requires more large-scale studies, personalized research, and the development of standardized treatment guidelines to optimize the clinical application of EMFs. It is important to establish clear protocols to ensure that EMF therapy is utilized effectively and safely in clinical practice.

## 5. Conclusion

In conclusion, musculoskeletal diseases can significantly impact a person’s quality of life, requiring early diagnosis and appropriate management. Conventional treatments, such as pharmacological interventions, physical therapy, and invasive procedures, have limitations that necessitate a constructive approach to exploring innovative modalities. EMF is a promising alternative that can stimulate tissue repair, reduce inflammation, and modulate pain signals. This non-invasive, painless, and safe therapy has minimal side effects and can be used alone or in combination with other therapies to provide personalized and comprehensive care for musculoskeletal diseases. With its historical roots and increasing scientific evidence, EMF therapy presents a promising avenue for improving musculoskeletal well-being and enhancing long-term health outcomes.
